# Does Electronic Monitoring Influence Adherence to Medication? Randomized Controlled Trial of Measurement Reactivity

**DOI:** 10.1007/s12160-014-9595-x

**Published:** 2014-02-27

**Authors:** Stephen Sutton, Ann-Louise Kinmonth, Wendy Hardeman, Dyfrig Hughes, Sue Boase, A. Toby Prevost, Ian Kellar, Jonathan Graffy, Simon Griffin, Andrew Farmer

**Affiliations:** 1 Behavioural Science Group, Primary Care Unit, Department of Public Health and Primary Care, Institute of Public Health, University of Cambridge, Cambridge, UK; 2 Primary Care Unit, Department of Public Health and Primary Care, Institute of Public Health, University of Cambridge, Cambridge, UK; 3 Centre for Health Economics and Medicines Evaluation, Bangor University, Bangor, UK; 4 Department of Primary Care and Public Health Sciences, King’s College London, London, UK; 5 Department of Primary Health Care Sciences, University of Oxford, Oxford, UK; 6 Institute of Public Health, University of Cambridge, Forvie Site, Robinson Way, Cambridge, CB2 0SR UK

**Keywords:** Measurement reactivity, Medication adherence, Electronic monitoring, Diabetes

## Abstract

**Background:**

Electronic monitoring is recommended for accurate measurement of medication adherence but a possible limitation is that it may influence adherence.

**Purpose:**

To test the reactive effect of electronic monitoring in a randomized controlled trial.

**Methods:**

A total of 226 adults with type 2 diabetes and HbA1c ≥58 mmol/mol were randomized to receiving their main oral glucose lowering medication in electronic containers or standard packaging. The primary outcomes were self-reported adherence measured with the MARS (Medication Adherence Report Scale; range 5–25) and HbA_1c_ at 8 weeks.

**Results:**

Non-significantly higher adherence and lower HbA_1c_ were observed in the electronic container group (differences in means, adjusting for baseline value: MARS, 0.4 [95 % CI −0.1 to 0.8, *p* = 0.11]; HbA_1c_ (mmol/mol), −1.02 [−2.73 to 0.71, *p* = 0.25]).

**Conclusions:**

Electronic containers may lead to a small increase in adherence but this potential limitation is outweighed by their advantages. Our findings support electronic monitoring as the method of choice in research on medication adherence. (Trial registration Current Controlled Trials ISRCT N30522359)

## Background

Accurate measurement of adherence is a prerequisite for rigorous research on the patterns, determinants and consequences of medication adherence. Several different methods are used to measure adherence, including self-reports, pill counts, pharmacy records, plasma drug levels and electronic monitoring. Each of these methods has advantages and disadvantages [1, 2]. However, electronic monitoring (e.g., by container caps that record the date and time of each opening) is widely recommended as the method of choice in research on medication adherence [3, 4]. Assuming that there is a one-to-one correspondence between opening the container cap and ingesting the prescribed dose of tablets, electronic monitoring can provide detailed, precise and objective data on daily adherence over an extended period. In research on medication adherence, electronic monitoring is used to analyze dosing patterns [5], as the primary behavioral outcome in trials of medication adherence interventions [6] and as the gold standard comparator for validating other measures of adherence [7].

A possible limitation of electronic monitoring is that it may influence adherence. Switching research participants to electronic medication containers may disrupt established routines and lead to reduced adherence. On the other hand, participants may increase adherence because they know that the researchers will be able to tell how adherent they have been and they wish to appear maximally adherent or because the medication container acts as a novel visual prompt or cue.

There is increasing interest in the possible reactive effects of measurement on behavior and other outcomes [8, 9]. Where they occur, reactive effects on behavior are usually positive, i.e., where the behavior is desirable, measurement produces an increase. Whether positive or negative, measurement effects may threaten the validity of conclusions that are drawn from research studies. For example, in an uncontrolled single-group study designed to estimate the effect of an adherence intervention, the use of electronic monitoring to assess adherence may lead to a biased estimate of the intervention effect.

Denhaerynck and colleagues [10] identified six studies that examined the possible effect of electronic monitoring on medication adherence [11–16]. The findings were inconclusive. Only two of the studies [15, 16] were randomized controlled trials. Elixhauser et al. [15] tested the effect of electronic blister packs compared with standard packaging over a period of 2–4 months in a sample of 93 psychiatric outpatients treated with lithium. Of four measures of medication adherence, only one showed a statistically significant difference between groups: the mean percentage of prescription refills obtained was 82 % in the electronically monitored group versus 69 % in the control group (*p* < 0.01). Wagner and Ghosh-Dastidar [16] compared monitoring using an electronic medication container with control in 120 HIV-positive patients on highly active antiretroviral therapy (HAART) over a period of 4 weeks. The adherence measure was the mean percentage of pills taken as prescribed based on a medication recall interview covering the previous 3 days. Adherence was high at baseline in both groups (electronic monitoring 93 %; control 92 %) and remained high at follow up with no difference between groups (91 % vs. 94 %, *p* = 0.73).

This paper reports a randomized controlled trial of the effect on adherence of dispensing medication in an electronic container that recorded the date and time of each opening (TrackCap, Aardex, Zurich, Switzerland) compared with standard packaging over an 8-week period, in a sample of patients with type 2 diabetes, using self-reported adherence as the primary behavioral outcome and HbA_1c_, a summary measure of recent glycemic (blood sugar) control, as the primary clinical outcome. As well as reporting the trial analysis, we examined the electronic monitoring data from the group allocated to using electronic containers to look for a pattern of daily adherence over the 8-week period that might indicate an effect of electronic monitoring. For example, a reduction in daily adherence over time would be consistent with a short-lived positive effect of electronic monitoring on adherence.

## Methods

### Participants

Patients were recruited from 13 primary care clinics in Oxfordshire, Buckinghamshire, Suffolk, Essex and Huntingdonshire (UK). Patients were eligible for inclusion if aged 18 years or over with type 2 diabetes of at least 3 months’ duration, able to give informed consent, currently taking any oral glucose-lowering agent and with a HbA_1c_ ≥ 7.5 % (58 mmol/mol). (An HbA_1c_ level of 7.5 % or above is widely used in clinical practice to indicate suboptimal blood glucose control which may in turn reflect inadequate adherence to glucose-lowering medication.) Those approached were deemed by their general practitioner to be appropriate for tight glycemic control and independent in medication taking.

### Design

**Fig. 1:**
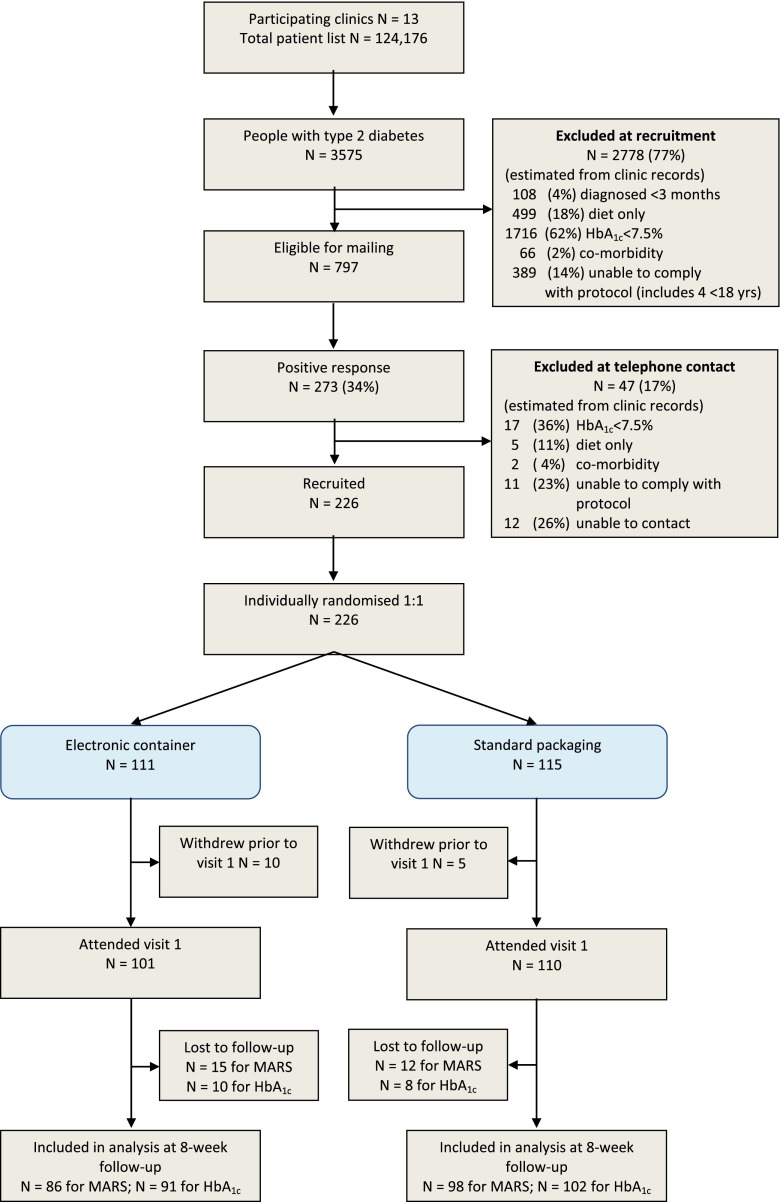
Flow of participants through trial

We used a parallel group trial design with 1:1 randomization to electronic container or standard packaging and follow up at 8 weeks (Fig. 1). Randomization was conducted before the baseline visit to the clinic (visit 1). Prior to visit 2 at 8 weeks, a second randomization was conducted to evaluate the effect of an adherence intervention delivered during that visit. The adherence intervention trial is reported elsewhere [17]; the present study focuses on the 8-week period up to and including visit 2.

Randomization of patients was carried out by the trial statistician independently of the trial co-ordination and clinic teams. A partial minimization procedure was used to dynamically adjust randomization probabilities to balance the baseline stratification variables (clinic, duration of diabetes, HbA_1c_ result from the clinic record and self-reported adherence). After the participant had been allocated to group, both the participant and the clinic nurse who saw the participant became unblinded to the participant’s group allocation. However, the laboratory staff who measured HbA_1c_ were blind to allocation. The London multi-centre research ethics committee reviewed and approved the protocol (06/MRE02/3).

### Measures

The primary behavioral outcome was self-reported medication adherence measured by postal questionnaire sent 1 week before visit 2 (8 weeks) using the Medication Adherence Report Scale (MARS) developed by Horne and colleagues [18]. We used a five-item version of the MARS that asked about “using your diabetes medicines in the last month”, with item responses scored on a 5-point scale from “always true” [1] to “never true” [5]. Scores are summed to give a score ranging from 5 to 25 with a higher score indicating higher self-reported adherence (Table 1). In a pilot study, we found that this version of the MARS had slightly better psychometric properties than alternative versions (e.g., a version that did not specify a time frame) and compared with the similar questionnaire developed by Morisky and colleagues [19]. In the present study, MARS at follow up correlated significantly (*r* = 0.27; *p* = 0.02; *n* = 81) with electronically measured adherence (using only the data from the last 30 days of the 8-week follow-up period, to match the time frame of the self-report measure). Cronbach’s alpha was 0.82 at baseline and 0.67 at follow-up. The primary clinical outcome was HbA_1c_ at 8 weeks.

**Table 1:** The Medication Adherence Report Scale (MARS) [14]

Many people find a way of using their medicines or tablets which suit them. This may differ from the instructions on the label or what their doctor has said. We would like to ask you a few questions about how you take your *diabetes medicines* (*tablets*).
The following statements show some ways in which other people have said they use their medicines.
For each of the statements below, please circle the number that best describes how you have taken your *diabetes medicines in the last month*.
1 I forget to take my diabetes medicines.
2 I alter the dose of my diabetes medicines.
3 I stop taking my diabetes medicines for a while.
4 I decide to miss out a dose of my diabetes medicines.
5 I take less of my diabetes medicines than instructed.

A secondary behavioral outcome measure, available only for participants in the group allocated to using electronic containers, was whether or not the correct number of doses of the main glucose lowering medication was taken as prescribed on each day over the 8-week follow-up period as measured by electronic monitoring.

### Procedure

The clinic nurse identified eligible patients registered with the clinic. Eligible patients were sent a letter from the clinic giving details of the trial, and a questionnaire asking about basic demographics, medication regimen, medication adherence (including the MARS) and beliefs about taking diabetes medicines. Responders were telephoned by the clinic nurse to arrange a recruitment visit at the clinic (visit 1). In advance of the visit, patients eligible and willing to take part were allocated to be dispensed medication in the electronic medication-monitoring device (TrackCap, Aardex, Zurich, Switzerland) or in standard packaging. At the visit, informed consent was obtained, clinical data were collected, blood was taken, and questionnaires completed. For those patients allocated to the electronic container, the nurse briefly explained its purpose and use and gave them an information sheet explaining how to use it. The patient was told that the container counts the number of times that the lid is removed, that they should only open and close the container at the time a tablet is to be taken, and that they should only remove the amount of medication required at the time. The nurse then referred them to the clinic dispenser or pharmacist to dispense their usual prescription for metformin or alternative oral glucose lowering agent in the device. Other medication was dispensed as usual, in standard packaging. For those allocated to the other trial arm, the clinic dispenser or pharmacist dispensed their medication as usual, in standard packaging. Patients in both trial arms who used a pillbox to organize their medications were able to continue to use this method during the trial, with the proviso that those in the electronic container arm had to use the electronic containers for their main oral glucose lowering agent. A second visit was arranged in 8 weeks.

In advance of the 8-week visit (visit 2), patients were sent a questionnaire (which included the MARS) from the coordinating center and were centrally randomized to the adherence intervention or a standard care visit. The patients were not told their intervention group allocation before they attended the visit. At the beginning of the visit, patients in both groups had blood samples taken for measurement of HbA_1c_.

### Analysis

The adherence intervention trial was planned to follow up 200 patients [20]. For the measurement effect trial, this sample size gave 80 % power to detect a difference of one point in the MARS self-reported adherence measure assuming a standard deviation of 2.5 (a small to medium effect size), and 80 % power to detect a 0.5 % difference in HbA_1c_ assuming a standard deviation of 1.25 % (two-sided tests, 5 % significance level).

Analysis of the trial data was by intention to treat. Outcomes were analyzed using analysis of covariance adjusting for their corresponding baseline value to improve precision. The missing indicator method [21] was used so that patients with a missing baseline value could be included. Since the MARS score was skewed, the results were checked using a Mann–Whitney *U* test.

An exploratory subgroup analysis was conducted to examine whether the effect of using the electronic container was different among participants who used a pillbox to organize their medications compared with the rest of the sample. This was done by extending the analysis of covariance to incorporate a test for interaction between trial arm and pillbox use (yes/no). An additional analysis (suggested by one of the reviewers) was conducted to examine whether the effect of using the electronic container was different among participants who were less adherent at baseline (as measured by self-report) compared with those who were more adherent; this involved testing the interaction between trial arm and baseline adherence (treated as continuous).

Generalized estimating equations (GEE), which take account of the dependence of the observations within individuals, were used to analyze change over time in the proportion of participants in the electronic container group who were adherent according to the electronic monitoring data on each day over the 8-week follow-up period. The analysis specified an autoregressive (order 1) correlation matrix and robust standard errors and tested linear and quadratic effects of time. Analyses were conducted in Stata 11 and PASW 16.

## Results

In the 13 participating clinics, 797 registered patients with type 2 diabetes potentially meeting the inclusion criteria were identified, of whom 273 responded as eligible and 226 were randomized (Fig. 1). The two groups were similar at baseline (Table 2). On average, participants were in their early 60s, had had diabetes for less than 10 years, were taking six medications daily and reported high medication adherence (scored 24 out of 25 on the MARS).

**Table 2:** Baseline characteristics of trial participants

	Electronic container (*n* = 111)	Standard packaging (*n* = 115)	All participants (*n* = 226)
% Male (*n*)	62.2 % (69)	67.8 % (78)	65.0 %(147)
Age (years)	63.7 (11.2)	62.8 (10.6)	63.2 (10.9)
IMD deprivation score (0–100)^a,d^	10.6 (6.3)	10.0 (6.8)	10.3 (6.6)
Duration of diabetes (years)	6.5 (4.8)	7.1 (5.4)	6.8 (5.1)
Weight (kg)^e^	94.5 (20.2)	97.8 (21.5)	96.2 (20.9)
% Treated with Metformin (*n*)^f^	70.0 (70)	77.3 (85)	73.8 (155)
Metformin daily dose (mg)^b,g^	1,767 (609)	1,695 (601)	1,728 (604)
Total number of medications taken/day^e^	5.6 (2.6)	6.0 (2.3)	5.8 (2.5)
Self-reported adherence (MARS,^c^ range 5–25)^h^	23.8 (1.8)	23.4 (3.0)	23.6 (2.5)
HbA_1c_ (%)^i^	8.31 (1.28)	8.35 (1.20)	8.33 (1.24)
HbA_1c_ (mmol/mol)^i^	67.33 (13.99)	67.77 (13.12)	67.55 (13.55)

Values are mean (SD) unless otherwise stated

^a^Index of Multiple Deprivation

^b^For those treated with Metformin

^c^Medication Adherence Report Scale

Number of missing values: ^d^2, ^e^15, ^f^16, ^g^72, ^h^25, ^i^32

Primary outcome data were available for 81 % and 85 % of those randomized for MARS and HbA_1c_, respectively. Missing outcome data were due to failure to return the questionnaire for MARS and non-attendance at visit 2 for HbA_1c_. In the main trial analysis, both outcomes showed a difference between groups, with higher adherence and lower HbA_1c_ in the electronic container group, but the differences were not statistically significant (*p* = 0.11 and *p* = 0.25, respectively; Table 3). This was confirmed by the results of the non-parametric test. There was no evidence that the effect of using an electronic container was different among the 23 % of participants who used a pillbox to organize their medications; the tests for interaction between trial arm and pillbox use were not statistically significant for self-reported adherence (*p* = 0.11) or HbA_1c_ (*p* = 0.71). There was also no evidence for an interaction between trial arm and baseline adherence on adherence at outcome (*p* = 0.93).

**Table 3:** Outcomes 8 weeks after randomization to electronic container or standard packaging

	Electronic container		Standard packaging		Estimated effect^a^ (95 % CI)	*p* value
	Baseline	8 weeks	Baseline	8 weeks		
Self-reported adherence^b^	23.8 (1.8)^c^	24.2 (1.1)^d^	23.6 (2.5)^e^	23.8 (1.9)^f^	0.4 (−0.1 to 0.8)^g^	0.11
HbA_1c_ (%) HbA_1c_ (mmol/mol)	8.31 (1.28)^h^ 67.33 (13.99)^h^	8.22 (1.30)^i^ 66.34 (14.21)^i^	8.36 (1.21)^j^ 67.87 (13.23)^j^	8.39 (1.16)^j^ 68.20 (12.68)^j^	−0.09 (−0.25 to 0.07)^k^ −1.02 (−2.73 to 0.71)^k^	0.25

Values are Mean (SD) unless otherwise stated

^a^Adjusted for baseline value

^b^Medication Adherence Report Scale (MARS)

Number of missing values: ^c^36, ^d^25, ^e^24, ^f^17, ^g^42, ^h^21, ^i^20, ^j^13, ^k^33

**Fig. 2:**
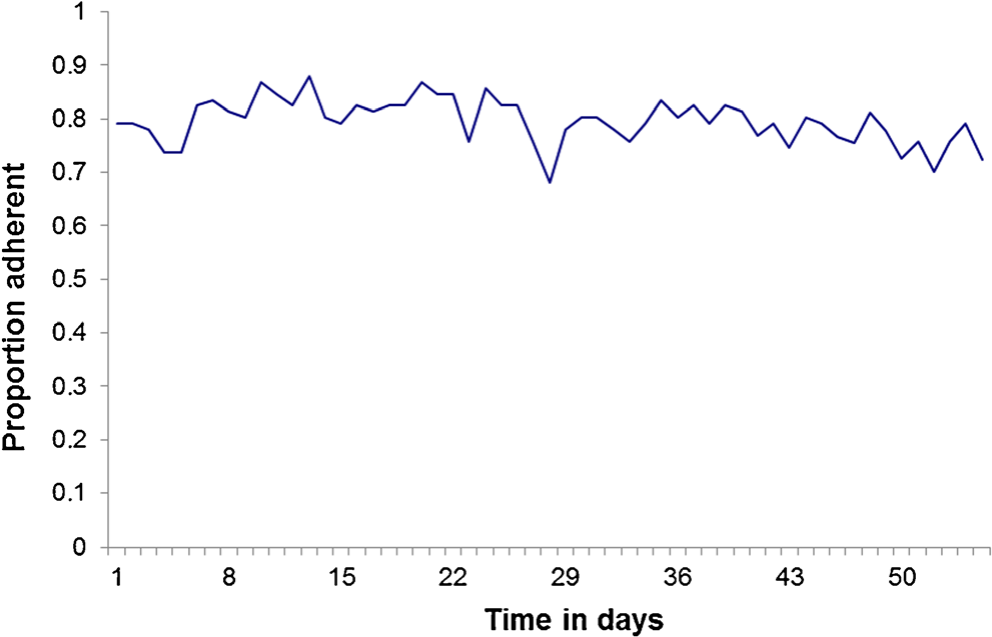
Proportion of participants in the electronic container arm taking their main oral glucose-lowering medication as prescribed on each day of monitoring

Within the electronic container arm, Fig. 2 appears to show a small reduction in the proportion adherent over time as measured by electronic monitoring. However, neither linear (*p* = 0.09) nor quadratic (*p* = 0.08) effects of time were statistically significant in the GEE analysis.

## Discussion

The trial analysis showed no statistically significant difference between electronic medication container and standard packaging on either outcome measure at 8 weeks. The confidence intervals around the estimates of the effect suggest that using an electronic container did not reduce adherence but may have increased it slightly.

A null effect may mask a negative effect in one subgroup offset by a positive effect in another subgroup. We explored the possibility that the effect differed in those participants who used a pillbox to organize their tablets and whose medication-taking routine may have been disrupted by switching to an electronic container for their main glucose lowering agent, but found no evidence for this.

The data from the electronic container group appeared to show a small initial increase in adherence that reduced gradually over time, which could be interpreted as being consistent with the trial results. However, there was no significant effect of time. This differed from the pattern observed in a study of HIV patients which suggested that adherence reduced over time and stabilized after 40 days [22].

Limitations of the present study concern the outcome measures and the high level of adherence at baseline, as reported in a previous trial [16]. HbA_1c_ is affected by a number of factors in addition to adherence to hypoglycemic medication, and 8 weeks may not be a sufficiently long period to observe a change in HbA_1c_ arising from a change in adherence. The MARS self-report measure referred to “diabetes medicines in the last month”, so this would not have detected an initial short-term increase or decrease in adherence. The MARS score correlated significantly (*r* = 0.27) with electronically measured adherence, providing some evidence of validity, but this was lower than the correlation of 0.42 reported for a ten-item version of the scale in a sample of asthmatic adults [23].

For the purposes of both the adherence intervention study and the measurement effect substudy reported here, we aimed to recruit a sample of patients with type 2 diabetes who showed suboptimal blood glucose control. The findings of the intervention trial [17] showed that it was possible to increase adherence in this group, though this was with adherence measured by electronic monitoring not by self-report. However, self-reported adherence was high at baseline in the present study and this may have reduced the chances of detecting an increase in adherence (though not of detecting a reduction in adherence).

Further investigation of this possible ceiling effect revealed that only 39 % of participants had the maximum score of 25 on the MARS at baseline, and that it was theoretically possible for the present study to have shown a statistically significant positive effect on self-reported adherence. An additional exploratory analysis showed that there was no evidence that the effect of using an electronic container was different in patients who were less adherent at baseline (as measured by self-report) compared with those who were more adherent. Nevertheless, future studies should aim to recruit less adherent patients.

Strengths of the present study include the trial design, with central randomization, concealment of group allocation, baseline comparability of groups and an acceptable proportion (>0.8) of those randomized providing primary outcome data.

Unlike other measures of adherence, electronic monitoring can provide detailed, precise and objective data on daily adherence over an extended period. The findings of this trial suggest that electronic containers may lead to a small increase in adherence but this potential limitation is outweighed by their advantages. Although other problems have been identified [10] and need further investigation, our findings support electronic monitoring as the method of choice in research on medication adherence.
